# Improving prediction of disease outcome for inflammatory bowel disease: progress through systems medicine

**DOI:** 10.1080/1744666X.2021.1945442

**Published:** 2021-06-28

**Authors:** Federica Giachero, Andreas Jenke, Matthias Zilbauer

**Affiliations:** a Witten/Herdecke University, Faculty of Health, Department of Medicine, Clinical Molecular Genetics and Epigenetics, Centre for Biomedical Education & Research (ZBAF), Germany; b Children´s Hospital Kassel, Department of Neonatology and Paediatric Gastroenterology, Klinikum Kassel, Mönchenbergstr, Kassel, Germany; c Department of Paediatrics, University of Cambridge, Addenbrooke’s Hospital, Cambridge, UK; d Department of Paediatric Gastroenterology, Hepatology and Nutrition, Cambridge, University Hospitals, Addenbrooke’s, Cambridge, UK

**Keywords:** Inflammatory bowel diseases, prognostic biomarker, predictive biomarker, systems biology, multi-omics, personalized medicine

## Abstract

**Introduction:** Inflammatory bowel diseases (IBDs) are lifelong conditions causing relapsing inflammation of the intestine. In the absence of a cure, clinical management of IBDs is extremely challenging since they present with a wide range of phenotypes and disease behaviors. Hence, there is an urgent need for markers that could guide physicians in making the right choice of the rapidly growing treatment options toward a personalized care that could improve the overall outcome.

**Areas covered:** In this review, the authors summarize existing biomarkers in IBD, discuss the challenges with the development of prognostic biomarkers and propose alternative options such as focusing on the prediction of the response to individual treatments, i.e. predictive biomarkers. The problems related to developing disease prognostic and predictive biomarkers in the field of IBDs are discussed including the difficulties in dealing with phenotypic heterogeneity particularly when performing studies in a real-life setting. The authors reviewed literature from PubMed.

**Expert opinion:** Systems biology provides potential solutions to this problem by offering an unbiased, holistic approach to adjusting for variation in larger datasets thereby increasing the chances of identifying true associations between molecular profiles and clinical phenotypes.

## 1. Introduction

Inflammatory bowel diseases (IBDs) are characterized by chronic relapsing inflammation of the digestive tract. IBD can affect patients in all age groups. In the past, the number of affected children and adolescent has been estimated to be between 20% and 25% of all IBD patients [1,2]. According to more recent data from a geographically well-defined cohort in Scotland this proportion might be much lower (1.5%) [3]. Nevertheless, the overall prevalence of IBD in the pediatric population has increased substantially over the last two decades in all industrialized nations [4–6].

The two major forms are Crohn’s disease (CD) and ulcerative colitis (UC). In UC inflammation is restricted to the large bowel (colon) mucosa and submucosa with disease commonly involving the rectum. Diarrhea, hematochezia, tenesmus, and defecatory urgency are classic symptoms of active UC [7]. CD in contrast is characterized by discontinuous regions of intestinal inflammation most frequently involving the terminal ileum and colon, even though it can affect any part of the gastro-intestinal (GI) tract from the oral cavity to the anus [8]. In CD, inflammation is transmural in nature and intestinal fibrosis, strictures, and fistula formation as disease complications are frequently seen [8].

In the absence of a curative treatment for IBD, patients face a lifelong requirement for treatment which includes a wide range of both medical and surgical interventions.

Following diagnosis, the course of disease varies widely even between individuals diagnosed with the same sub-entity (i.e. UC or CD). These differences apply to distribution and severity of intestinal inflammation, the presence of extra-intestinal manifestations, development of complications (e.g. strictures) as well as the impact of disease on quality of life to name just a few [9]. Unfortunately, disease severity and distribution at diagnosis do not correlate with longer term outcome making it difficult to tailor initial treatment. Later on, a lack of correlation between clinical phenotype and routinely used serum markers with mucosal healing complicates matters further [10].

**Figure 1.**
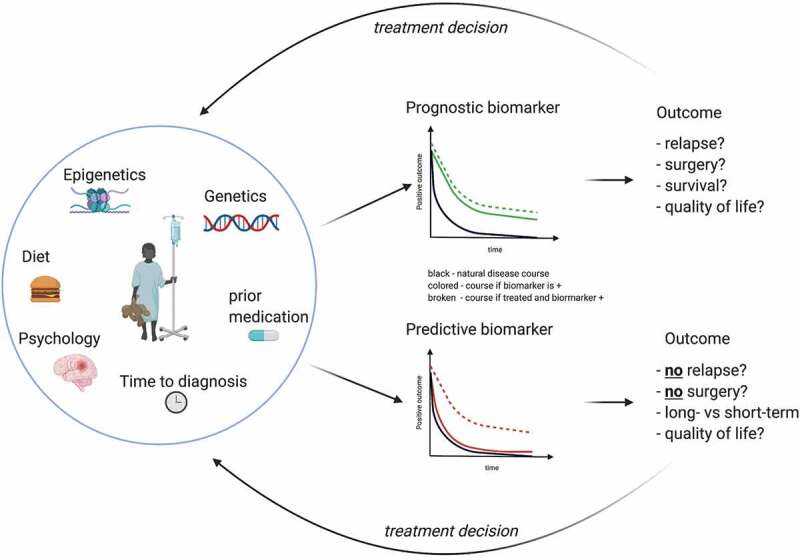
Difference between prognostic and predictive biomarkers.

The development of disease prognostic biomarkers is even more complicated by the absence of a widely accepted and/or validated disease outcome score, particularly since defining disease outcome in IBD is far from trivial given the wide range of organic and non-organic factors contributing to patient health and wellbeing [11]. Indeed, subjective disease-specific quality of life has been shown to correlate with physical activity and disease activity in patients with IBD and might even have reciprocal impact of these factors (Figure 1) [12].

In the past several parameters have been proposed as potential predictors of the long-term outcome and of the response to therapy in CED – in many cases these terms were used interchangeably even though prognostic and predictive biomarkers focus on two different aspects entirely. Whereas a prognostic biomarker is a marker that provides information on the likely course of the disease in an untreated individual, predictive biomarkers are defined as markers that can be used to identify subpopulations of patients who are most likely to respond to a given therapy.

In contrast to disease prognostic biomarkers, predictive biomarkers provide information on the likely response to individual treatment interventions (Figure 1). For a personalized clinical management these are equally important since the currently available medical treatment options are only effective in approximately 40–60% of patients. However, defining treatment targets represents – similarly to disease outcome – a major challenge, that has been started to be addressed recently by the Selecting Therapeutic Targets in Inflammatory Bowel Disease (STRIDE) initiative aimed to define standardized treatment goals in adults [13] and children [14]. At a very basic level, response to treatment can be assessed by evaluating disease activity before and after treatment. Although an initial response to treatment does not guarantee prolonged efficacy as many patients lose response to treatment over time, stratification of treatment according to predicted response would undoubtedly be of major benefit to the treating physician.

**Figure 2.**
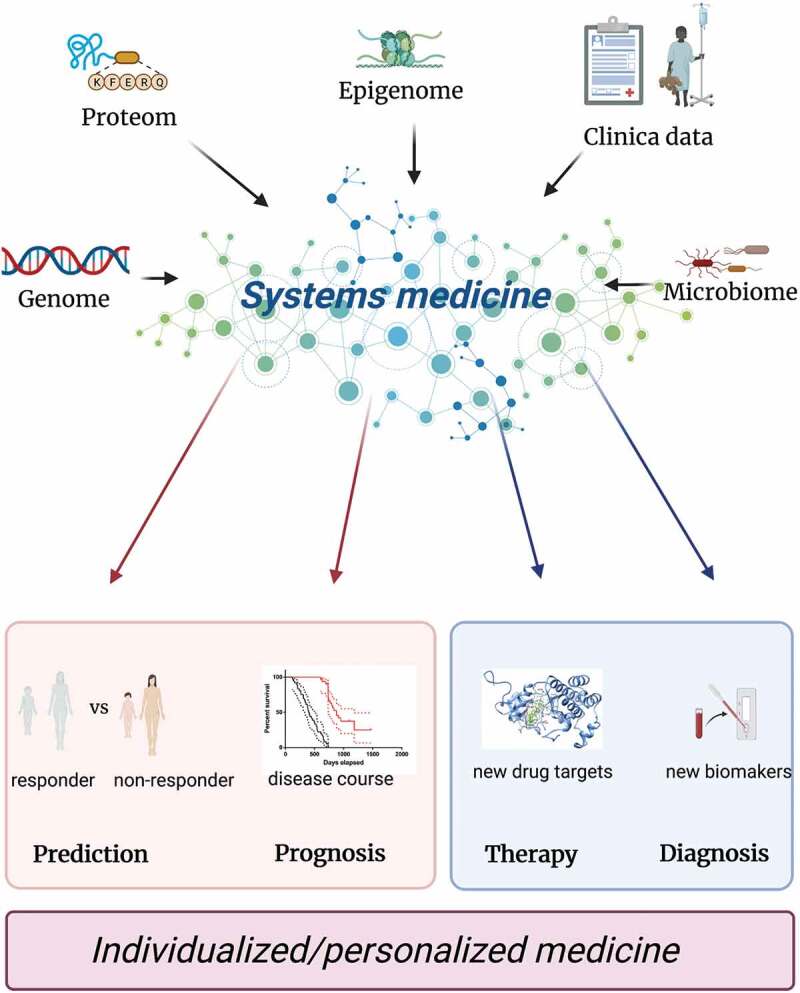
Systems medicine approach.

Taken together, in order to make real progress in the development of disease prognostic and/or predictive biomarkers in IBD, both disease heterogeneity and complexities of large omics datasets must be addressed in structured and unbiased way. Systems medicine offers a sophisticated approach to investigate not only complex molecular and cellular interactions but also allows for a holistic, systematic, and unbiased analysis of integrated high-throughput omics with clinical datasets (Figure 2) [15]. Major progress has been made in other conditions using this approach and despite differences and unique challenges specific to IBD, plenty of insight can be gained by reviewing approaches taken in other areas.

Based on recent technological developments it is nowadays possible to integrate high-throughput omics (including genome and epigenome, transcriptome, proteome, metabolome, and microbiota to cite the most important aspects) with clinical datasets. Analysis of these multi-layered sets of information might open new perspective creating a circular and iterative process between clinical setting and computational pathogenetic models, allowing to develop new models for diseases prognosis and prediction of response to therapies.

In this review we summarize current progress in the field of disease prognostic and predictive biomarkers in IBD. Furthermore, we will highlight major limitations, challenges, and opportunities as well as potential benefits of applying systems biology approach.

## 2. Existing biomarkers in IBD

### 2.1. Disease prognostic biomarkers in IBD

Broadly speaking, disease prognostic biomarkers aim to predict the likelihood of a clinical outcome such as disease recurrence or progression in patients with a specific underlying medical condition [16]. Their importance and benefit include the provision of information to the patient regarding their expected future life (i.e. disease prognosis). Importantly, the ability to predict specific disease complications and overall outcome is the basis of a personalized therapy approach. Tailoring treatment by providing the most potent medications to patients who are expected to experience a severe disease course would limit exposure to potential side effects to patients with mild diseases. Importantly, by treating patients with a predicted severe disease more aggressively early on, the natural course of the disease might be changed thereby improving long-term outcome. This applies for both – newly diagnosed patients and for patients during the disease course in case of severe complications requiring hospitalization for urgent rescue therapy [17]. However, current data comparing generalized top-down (i.e. starting with the most potent treatment first) with step-up (starting with milder treatments) therapeutic approaches are insufficient to fully support this assumption. Whilst top-down approaches have been shown improved short-term prognosis possibly related to the more aggressive immunosuppression resulting in faster control of the inflammation [18], long-term outcome does not appear to be superior compared to a step-up approach [19]. Taken together, there is insufficient evidence in support of any currently available medical treatment being capable of altering (i.e. improving) the natural course of disease. Future studies are still needed to shed further light on this important issue and therefore the final verdict on the potential value of disease prognostic biomarkers in this respect remains to be determined.

Nevertheless, documentation of disease behavior and outcome aiming to understand the natural course of disease forms a fundamental first step in the development of disease prognostic biomarkers in IBD [20,21]. Equally important is the ability to quantify disease outcome in a reliable, clinically relevant, and reproducible manner. In the absence of a widely accepted and validated outcome score in IBD, existing studies have used a wide range of outcome measures thereby limiting comparability as well as reproducibility [22].

In the following, we will briefly summarize some of the existing evidence on the development of disease prognostic biomarkers in IBD. In order to guide the reader, we will distinguish between clinical and serological markers in UC and CD.

### 2.2. Ulcerative colitis

#### 2.2.1. Clinical markers in ulcerative colitis

Due to its continuous involvement of the rectum and colon, disease progression in UC is most commonly defined by proximal disease extension which has been reported to be associated with a more severe disease course [23]. In a recent study the rate of progression from left-sided UC to extensive colitis ranged from 21% to 34% and about two-thirds of patients required hospitalization within 10 years of diagnosis [24]. Furthermore, the cumulative 10-year risk for surgery in UC patients has been reported to be around 15% [25]. Based on these fairly well-defined criteria, several studies have tried to identify clinical parameters which correlate with future disease progression including the risk for surgery in UC. Primary sclerosing cholangitis, requiring steroids or immunosuppression and being a nonsmoker were found to be associated with proximal disease extension. However, it is worth noting that positive and negative predictive values for these parameters were very low thereby limiting their potential clinical application [23,26,27]. Interestingly, evidence from an early study dating back to the ‘70s on children with UC showed that if duration of symptoms prior to formal diagnosis was more than 24 months, patients were found to have a significantly higher probability for a complicated disease course [28]. On the other hand, a shorter time from the onset of symptoms to disease particularly when presenting as acute severe colitis, has been shown to be associated with a high risk for a failure to respond to treatment with intravenous steroids [29]. One retrospective cohort study suggests that children between 5 and 10 year of age at diagnosis are more prone to a severe disease course than adolescents. However, this still needs to confirmed by other studies and might thus only reflect a regional effect or be a surrogate parameter for other underlying genetic or epigenetic factors yet to be defined [30]. Whilst clinical parameters and disease presentation at diagnosis has been found to be of limited value in predicting longer term prognosis, numerous studies have shown that disease progression and early response to treatment within the first 3 months from diagnosis might be more valuable. For example, disease activity in children diagnosed with UC measured by the Pediatric Ulcerative Colitis Activity Index (PUCAI) at three months was reported to be a strong predictor of 1 year sustained steroid-free remission [31,32]. Similarly, failure to achieve remission at 3 months implied an 80% risk for the requirement of treatment with biologics or major surgery within 18 months [33] further suggesting that clinical disease behavior during the first 3-month post diagnosis is indicative of longer term prognosis in UC patients and therefore likely to be highly valuable to guide future treatment and patient management.

#### 2.2.2. Serological markers in ulcerative colitis

Numerous serological markers have been tested for a potential correlation with disease outcome in UC. Among them are perinuclear antineutrophil cytoplasmic antibodies (pANCA) which have been suggested to be moderately prognostic of frequent relapses and a more severe course of the disease. In contrast, no correlation with outcome was found for the Interleukins (IL) 1β, IL6, IL15 and the serum inflammatory marker C-reactive protein (CRP) [34]. Subsequent studies challenged this hypothesis and showed pANCA not to be a reliable predictor of overall disease prognosis [35], although high pANCA levels seemed to indicate a higher risk of chronic pouchitis – an overall relatively rare complication in children – following ileal pouch-anal anastomosis in patients post colectomy [36]. Similar findings have been reported for elevated serum anti-flagellin antibodies (anti-CBir1) [37]. Furthermore, the serum granulocyte macrophage colony-stimulating factor auto-antibody (GM-CSF Ab) might be a promising candidate in identifying CD and UC patients at risk of disease relapse at an early stage [38]. However, currently confirmatory studies are still lacking. Interestingly, for very well-defined outcomes with major morbidity such as acute severe colitis (ASC) more reliable prognostic biomarkers are available. For example, the combination of hemoglobin, CRP and disease extension at diagnosis reliably predicted the risk of ASC within 3 years in different populations (Cambridge, Oxford and Uppsala) [39]. A recent study by Orlanski-Meyer *et al*. extensively investigated all available scientific data on possible prognostic biomarkers in children with ulcerative colitis. They identified markers for almost all relevant prognostic questions. For example, hypoalbuminemia at diagnosis and disease severity at onset, evaluated by PUCAI or endoscopic assessment, seem to predict future ASC [32]. It is worth noting that the odds ratio for almost all disease predicting parameters identified is low, thereby limiting their potential clinical value. In addition to serological markers, mucosa derived molecular signatures have also been tested for their disease prognostic value. For example, mucosal TNF-alpha expression combined with histological disease activity scores at the point of diagnosis have been reported to be predictive of a severe outcome in UC with a positive and negative predicate values of 0.89 and 0.87 respectively [40]. However, such parameters are very difficult to implement in routine clinical practice and results are still pending validation in larger, independent patient cohorts.

## 3. Crohn’s disease

### 3.1. Clinical markers in Crohn’s disease

The first population-based data on the clinical course and possible prognostic factors for patients with CD came from Olmsted County. In this cohort, patients with ileal and ileocolonic disease distribution at diagnosis were found to be five to seven times more likely to experience future complications such as the development of fistula or strictures when compared to those with isolated colonic disease [41]. The most comprehensive data on clinical predictors for a severe disease course in CD comes from two large tertiary-center studies. Among other factors an initial requirement for steroids, age at diagnosis of less than 40 years, ileocolonic involvement, or the presence of perianal disease at diagnosis were independently associated with disabling disease [42,43]. Furthermore, disease location, age at diagnosis, penetrating disease at presentation and smoking have all been associated with a more complicated course (Table 1) [44–47]. However, many of these risk factors have been identified in retrospective studies and lack validation in larger, prospective cohorts. Importantly, the definition of severe, disabling disease varied vastly amongst studies ranging from the prescription of more than two courses of oral steroids, further hospitalization after diagnosis for flare-up or disease complications, need for surgical intervention (e.g. for perianal disease) or the requirement for and escalation to immunosuppressive therapy. Furthermore, the majority of studies included all patients with Crohn’s, and many did not take any measures to account for phenotypic heterogeneity. Together, the complexity of CD phenotypes combined with the lack of a universally accepted, validated, and quantifiable outcome measure highlights the major limitations of existing evidence.

**Table 1.** Risk factors for a more complicated disease course in Crohn’s disease and ulcerative colitis

**Crohn’s disease**	Ref	**Ulcerative colitis**	Ref
Delayed diagnosis	[58]	Delayed diagnosis	[28,44]
Black and South Asian ethnicity	[58]	Long-standing disease duration (>10 years)	[32,44]
Male gender	[58]	Male gender	[32,44]
Growth impairment at diagnosis	[50,58]	Young age at diagnosis	[32,44]
Younger age at diagnosis (higher risk for growth impairment)	[58]	Family history for IBD	[32]
Older age at diagnosis (e.g. > 13 years; higher risk for complications and for surgery)	[58]	Disease extension at diagnosis and over time	[32]
Extensive disease (pan-enteric inflammation) or deep colonic ulcers	[50]	Disease severity at diagnosis (assessed clinically through PUCAI score 65 or higher, or through endoscopy)	[32]
More active disease at diagnosis or over time	[58]	High histological inflammation score	[44]
Stricturing disease (demonstrated by endoscopic or radiological examination) at diagnosis, obstructive signs/symptoms, pre-stenotic dilatation	[50,58]	Neutrophilic inflammation of stomach and duodenum	[32]
Penetrating disease (bowel perforations, intra-abdominal fistulae, inflammatory masses, and/or abscesses at any time in the course of the disease and not as result of surgical complications)	[50,58]	Primary sclerosing cholangitis	[32]
Perianal disease	[50,58]	C. difficile infection	[32]
Small bowel disease location (higher risk of growth impairment, stricturing/penetrating complications, multiple surgeries)	[44,58]	Extra-intestinal manifestations	[24]
Ileal or ileocolonic disease location (higher risk of surgery, complications, progressive disease, disabling disease)	[44]	Elevated CRP at diagnosis	[24]
Colonic disease location (risk of permanent stoma)	[44]	Low hemoglobin (<10 g/dl) at diagnosis	[65]
No clinical remission (PCDAI > 5) 12 weeks after start of induction therapy	[50]	Low serum levels of vitamin D	[65]
No biochemical remission (CRP > 20 mg/l, fecal calprotectin > 400 µg/g) 12 weeks after start of induction therapy)	[50]	Erythrocyte sedimentation rate (ESR) ≥30 mm/h	[27]
Antibodies against OmpC (E.coli outer membrane porin C) and against CBir1 (antiflagellin)	[50,58]	Hypoalbuminemia	[32]
Antibodies against ASCA (Saccharomyces cerevisiae)	[50,58]	Frequent disease flares (> 3 per year) and frequent hospitalization	[44]
Low variety in microbiome	[59]	Steroid dependence or resistance	[44]
Presence of NOD2/CARD15 variants	[58]	PUCAI score at day 3 and at 3 months	[29,32]
Smoking	[44]	Antibodies against ASCA (Saccharomyces cerevisiae) and ANCA (antineutrophil cytoplasmic antibodies)	[32]
		Low variety in microbiome	[59]
		No smoking	[44]

Nevertheless, novel imaging techniques might provide alternative approaches. Although not a purely clinical parameter, complex MRI scoring systems quantifying the amount of CD activity and severity in each segment of the bowel have been used to predict the future disease course. Higher scores on the so-called Lémann Index for example have been shown to be associated with an increased risk of surgery and hospitalization [48]. For a comprehensive review on clinical predictors in CD we recommend reading Peyrin-Biroulet et al. [49]. For a state-of-the-art guidance on the medical treatment and long-term management of children and adolescents with CD we recommend reading the most recent ECCO-ESPGHAN guideline update [50].

Taken together, there is currently no convincing evidence for clinical parameters at the point of diagnosis to predict future disease outcome in CD patients.

### 3.2. Serological markers in Crohn’s disease

Over the last decades, several attempts have been made to identify serological markers prognostic of more aggressive phenotypes. Among those one of the most promising have been antibodies against Saccharomyces cerevisiae antibody (ASCA) [51–53]. Since then several studies have shown ASCA to be associated with a more complicated disease course (albeit definition of ‘complicated’ varies as outlined above). A meta-analysis including 24 studies with almost 4200 pediatric and adult patients showed significant association between the ASCA-positive status and higher risk of early-onset disease, ileal involvement, complicated disease behavior, perianal disease, and risk for surgery. Odds ratio however were again only moderate varying between 1.49 and 2.25 [54]. In a recent pediatric cohort significant differences in gut microbiome composition where found in ASCA-positive and ASCA-negative patients with CD implicating the microbiome in the underlying pathophysiological mechanism [55]. Similarly, other antibody responses against CD-related bacterial molecular patterns have been associated with a more complicated disease course such as Escherichia coli outer membrane porin C [56] or flagellin Fla-X [57]. Positive and negative predictive values were however very low. Aiming to provide clinical guidance an expert panel reviewed existing evidence on disease prognostic biomarkers in Pediatric CD. Even though the association between antimicrobial serology and specific future events such as penetrating disease or risk for surgery was acknowledged, the panel was unable to identify a single prognostic marker that could reliably predict future disease activity or the number of relapses at the point of diagnosis [58]. Importantly, the panel highlighted the need for targeted longitudinal studies in order to further characterize prognostic factors in pediatric CD.

Another field entirely and by definition not a serological marker is the characterization of the microbiome in IBD patients for disease prognosis. Whereas data is still rare compared to genomic, epigenomic, transcriptomic, proteomic, and metabolomic data, first results seem to be promising. In a recent study on 143 patients for example IBD phenotype and the risk of surgery could be predicted on the basis of 16S and 18S rRNA sequencing data [59].

### 3.3. Disease predictive biomarkers

In contrast to disease prognostic biomarkers, predictive biomarkers aim to provide information on the anticipated response of a patient to a specific treatment intervention [60]. In IBD, a range of existing treatments as well as the increasing number of novel therapeutics combined with the limited treatment effect (ranging between 40–60%) highlights the urgent need for the development of disease predictive biomarkers [61]. Similar to the lack of reliable outcome measures in IBD, there continues to be controversy about the common definition of remission [62]. This is important as an entering remission represents a key target to determine response to treatment. However, to date no reliable noninvasive biomarker has been identified and mucosal healing assessed through endoscopy is still the most reliable and valid endpoint for remission and response to treatment [22]. Hence, the need for repeat endoscopy requiring substantial resources and time, as well as being unpopular among patients, substantially impacts on the development of clinical trials aiming to develop disease predictive biomarkers.

Several clinical and biological parameters have been investigated as potential predictive biomarkers so far. Most of them belong to three main categories: (i) clinical features [61] such as disease extent at diagnosis and early clinical response to treatment, (ii) routine laboratory tests [61] including FC, CRP, hemoglobin (Hb) erythrocyte sedimentation rate (ESR), vitamin 25-OH D, antineutrophil cytoplasmic antibodies (ANCA), and (iii) components of the immune cascade such as cytokines and immune cells [63,64].

In the following we briefly summarize existing evidence on disease predictive biomarkers in IBD.

### 3.4. Ulcerative colitis

Among the clinical features investigated in Pediatric UC patients, the short-term corticosteroid response is one of the first successfully identified predictors: in 2010 the OSCI study [29] showed that the Pediatric Ulcerative Colitis Activity Index (PUCAI) at day three of steroid therapy predicted therapy response up to one year later. In a more recent study, the clinical response to steroids treatment at three months [33] confirmed to offer reliable information about responsiveness to steroids in later disease course. On the contrary poor response at three months correlated with a higher probability of steroids failure and need of biologics or surgery at 18 months. The relatively recent PROTECT Study [65] (Predicting Response to Standardized Pediatric Colitis Therapy) followed an inception cohort of Pediatric UC patients, who received standardized treatment with mesalazine or steroids. Perhaps not surprising yet still of interest, the study found that patients presenting with mild disease at diagnosis and a good response to first line treatment had a higher chance of steroid-free remission at one year. Additionally, low baseline hemoglobin, low serum vitamin 25-OH D and low eosinophil count in rectal biopsy showed a correlation with non-response to steroid/mesalazine requiring escalation to anti-TNFα treatment. Despite several of the assessed parameters not reaching statistical significance, the approach of the whole study is a first example of a system medicine approach in this field. Pre-defined criteria for therapy response and escalation, prospective approach, extensive patients’ characterization at diagnosis including clinical, laboratory and histology data, but also rectal transcriptome, microbiome and high-density DNA genotyping are all major strengths and likely to yield important evidence.

A more invasive study analyzing genome wide transcriptional signatures from pre-treatment colonic mucosal biopsies found around 200–400 differentially expressed mRNAs in patients responding to anti-TNFα treatment compared to non-responders. The top five differentially expressed genes (osteoprotegerin, stanniocalcin-1, prostaglandin-endoperoxide synthase 2, IL-13 receptor α2 and IL-11) in both cohorts were able to separate responders from non-responders with 95% sensitivity and 85% specificity were [66]. However, despite the no doubt promising findings, so far this data has not yet been translated into a clinically applicable test.

### 3.5. Crohn’s disease

In CD shorter disease duration [67], and colonic involvement, instead of isolated ileal disease, has been moderately associated with better treatment response both in the short- and long-term in general [68,69]. Also, infliximab levels at week 14 have been reported to be significantly associated with 54-week efficacy of anti-TNFα treatment [70] as well as failure to achieve remission after induction [71]. However, the positive predictive value was relatively low. Similarly, a recent review could identify several factors such as severe disease, *a priori* anti-TNF exposure to be associated with non-respond to vedolizumab: and ileocolonic disease, no prior surgery and uncomplicated phenotype with better responses to ustekinumab. Still, positive and negative predictive values were disappointing [72]. More recently, the presence of ≥ 20 of mTNF-positive cells on confocal laser endomicroscopy was associated with a better response to adalimumab with 84.6% sensitivity and 91.7% specificity [73]. However, this technique is rather invasive and also not widely available.

A growing number of studies are now emerging focusing on predicting treatment response in CD patients including genetic polymorphisms of apoptosis regulators [74] and immunomodulators [75], as well as several cytokines [76]. Among the most promising candidates available is oncostatin M (OSM) [77]. In both, CD and UC, high OSM expression in pre-treatment mucosal biopsies of IBD patients was strongly associated with an early need for biologic therapy and lack of response to anti-TNFα treatment [78]. A later study investigated the predictive value of serum OSM and came to a similar conclusion [79].

Last and similarly to disease prognosis, microbiome data has been used to predict treatment response particularly to anti-TNFα. However, results were negative so far [80].

#### 3.5.1. Systems medicine approach for outcome prognosis

Systems medicine is a fast-evolving interdisciplinary field aiming to implement systems biology approaches in medical concepts, research and practice. Its principle is to analyze diseases in a holistic manner, by integrating systems biology platforms along with clinical parameters, for the purpose of understanding disease origin, progression, exacerbation, and remission. This involves iterative and reciprocal feedback between clinical practice and assessment with computational, statistical, and mathematical multi-scale analyses and modeling of pathogenetic mechanisms, disease progression and remission, disease spread and cure, treatment responses, and adverse events (Figure 2) [81]. One of the first example how a systems medicine approach can successfully influence disease treatment and even cause a paradigm shift is the discovery of viral dynamics in HIV-1 pathogenesis leading to modern combinatorial treatments [82].

In the field of outcome prognosis Salvucci et al. used a systems biology signature by linking APOPTO-CELL, a mathematical model of caspase activation resulting in apoptosis, with protein expression data and a machine learning approach to successfully identify prognostic biomarkers in stage III colorectal cancer patients who have an extensive genomic, epigenomic, and molecular interpatient heterogeneity [83].

Given the above, the application of systems medicine approaches might ultimately hold the key to unlocking the door to the successful development of both disease prognostic and predictive biomarkers in IBD.

#### 3.5.2. Systems medicine approach to biomarkers in IBD

A first attempt into this direction has been made by Khan et al. several years ago [84].

By combining clinical and epidemiological parameters in newly diagnosed patients with UC they achieved a reasonably well prognosis on the disease course with an area under the receiver operator curve of 0.71 [95% CI, 0.67–0.76]. However, the outcome focused on corticosteroid utilization which does not necessarily mean insufficient response to the primary medication, since this can also be influenced by other factors as for example adherence to medications which has been identified as a key factor in maintaining disease remission in UC [85].

A more recent study used a computational miRNA-based algorithm in UC patients after restorative proctocolectomy to predict the development of pouchitis. Interestingly, the combination of 11 miRNA expression profiles and 3 biological/clinical factors showed an accuracy of 88% (area under the curve = 0.94) to the prediction of recurrent or chronic pouchitis [86]. Similarly, in Crohn´s disease several tools have been developed combing parameters such as patient and disease characteristics, immune responses to ASCA, CBir1, and pANCA, and NOD2 status. One such example is the Personalized Risk and Outcome Prediction Tool (PROSPECT) aims to categorize patients into low risk, intermediate risk, or high risk for developing complications from their Crohn’s disease [87,88]. Even though many variables were considered, the Harrell’s C was only 0.73 and PROSPECT could not establish itself in clinical care. Similar models have been developed in several different regions worldwide with similar impact [89,90]. All models showed mediocre performance at best with sensitivities and specificities below 70%. Another more biological initially promising approach – in both UC and CD – identified analogous CD8 + T cell transcriptional signatures that discriminated patients into two otherwise indistinguishable subgroups which subsequently experienced very different disease courses. Namely, a substantially higher incidence of frequently relapsing disease was experienced by those patients with elevated expression of genes involved in antigen-dependent T cell responses, including signaling initiated by both IL-7 and TCR ligation – pathways [91]. However, whereas this seemed to be promising at first sight a larger more detailed validation cohort failed to reproduce the results [92]. Quite similar in its approach, the predictive value of proteome analysis prior therapy initiation has been studied for the need of intensified treatment in inflammatory bowel disease. In this study, the authors identified in 66 patients a combination of five core proteins ITGAV, EpCAM, IL18, SLAMF7, and IL8 which define a high-risk subgroup in IBD (HR 3.90, CI: 2.43–6.26) [93]. Also, minor but significantly different expression profiles in FCGR1A, FCGR1B, and GBP1 were found between responders and non-responders to anti-TNFα therapy two weeks after initiation of treatment in an observational, prospective cohort of Pediatric patients [94]. Even though both studies seem promising, larger trials are required that confirm these findings.

In another study on the Pediatric Risk Stratification Initiative (RISK) cohort, funded through the Crohn’s and Colitis Foundation, a model that combines gene expression, microbial abundance and clinical features, such as age at diagnosis and PCDAI score, was able to more accurately predict steroid-free and surgery-free remission than a model based on clinical parameters alone (area under the receiver operating curve 0.760 versus 0.705) [95].

Focusing on a different aspect of IBD entirely is the sequential analysis of fecal bacterial and fungal profiles. Interestingly, these seem to differ significantly between response groups before start of treatment with infliximab. More specifically, non-responders showed lower abundances of short chain fatty acid producers and bacterial taxa composition were able to predict the response to infliximab treatment in both CD and UC patients with an AUC above 0.8 [96]. Even though all these examples are no true system medicine approaches in its general sense, they demonstrate the value of focusing on large complex biological networks in the search for prognostic and predictive biomarkers. This is further supported by a very recent study which combined proteomic, metabolomic, and microbial data to successfully identify a pro-inflammatory state in quiescent IBD that predisposes to clinical relapse. More specifically, the proteins interleukin-10, glial cell line-derived neurotrophic factor, and T-cell surface glycoprotein CD8 alpha chain in combination with the metabolomic markers propionyl-L-carnitine, carnitine, sarcosine, and sorbitol and an increased abundance of certain bacteria were associated with relapse in multivariable models [97]. Also, even though not in the field of biomarkers but of new therapeutics Lloyd et al. used a systems medicine approach analyzing NF-κB signaling during GI tract inflammation to identify new candidate drugs. Among several in routine use for IBD, most prominently the corticosteroids, the authors also identified clarithromycin as potential therapeutic agent. Importantly, after this in-silica analysis the authors were able to demonstrate clarithromycin induced suppression of TNF-induced NF-κB (p65) nuclear localization in human intestinal organoids, demonstrating the whole potential of systems medicine [98].

## 4. Conclusions

Inflammatory bowel diseases cover conditions with wide ranging phenotypes that affect individuals to a varying degree. In this review, we have summarized the current progress in the search for prognostic and predictive biomarkers and the potential role of systems medicine in this race. It has become clear that prognosis of disease behavior and long-term clinical outcomes as well as the prediction of the individual treatment response to a specific therapy in patients with IBD is still very challenging. The main reasons for this are the high genomic, epigenomic, and molecular inter-patient heterogeneity and the unresolved problem how to define disease outcome. Combined with a rapidly increasing repertoire of therapeutic options, systems medicine provides a very promising approach to address the urgent need for a personalized treatment in IBD.

## 5. Expert opinion

The development of molecular markers capable of predicting outcome or response to treatment in patients with IBD is far from trivial for a number of reasons. Perhaps the most important obstacle is the fact that despite the wide range of fundamentally different phenotypes observed even within IBD subtypes (i.e. UC and CD), our knowledge of the underlying mechanisms responsible remains limited. Thus, one important challenge in the near future is the development of an unbiased data-driven analysis strategy integrating genomic, epigenomic, transcriptomic, proteomic, metabolomic, and microbiome information to build a comprehensive molecular map of IBD. To achieve this an appropriate and standardized data collection at diagnosis is critical. Taking into account the complexity and the costs of such a comprehensive approach, we expect that this will not be implemented on a large scale before the next five to ten years. To achieve this goal, it is essential that scientific societies support the systems biology approach through their recommendations. In this way it will be possible for the national health care providers to introduce such procedures in the routinely provided care.

Considering the expected diversity of IBD and assuming that a valid disease prognostic biomarker is relevant to disease pathogenesis, as variations could therefore explain differences in disease behavior, it seems unlikely that a single marker could be applied to all patients. In our opinion it is therefore of critical importance to recognize disease heterogeneity and develop computational tools to account for them. Secondly, with regards to the development of disease prognostic biomarkers, the development of reliable, clinically meaningful and validated outcome measures is a pre-requisite. The frequently taken approach of identifying a molecular marker followed by the search for any clinical parameter that shows a significant association is likely to fail. Importantly, as disease outcome cannot be fully defined by a single parameter and will at least in part be determined by patient specific/subjective parameters, a suitable score should consider the patient’s perspective. Last but not least, with IBD frequently being diagnosed early in life, we should not underestimate the fact that disease outcome is not limited to the first 5 years after diagnosis but in most patients will relate to several decades. This is yet another reason for considering shifting our focus on the development of predictive markers that are able to provide guidance on the suitability of specific treatment options at any given time. In this endeavor to define the individual patient within a population of complex genetic and epigenetic heterogeneity system medicine might be the optimal tool to achieve success. This will require a very close cooperation between clinical and research facilities in order to generate and integrate large amount of clinical information with multi-omics datasets. The coordination of such a network of clinicians, scientists and bioinformaticians, scattered in different locations will be very challenging. One main prerequisite will be that the care of patients with highly complex diseases, such as IBD, needs to be centered in highly specialized facilities where all aspects of the holistic systems medicine paradigm can be addressed. On the other side high quality clinical care needs to be accessible for all patients even in more remote locations. Thus, in the long-term the aim should be to design diagnostic and therapeutic algorithms for the several sub-entities of CED and to implement automatization of data interpretation (especially regarding the several and complex multi-omics datasets) thus translating research results from the bench into benefits for the patient at the bedside.

Considering the obstacles in this endeavor, in the mid-term future (e.g. in the course of the next five years) it seems reasonable to achieve relevant improvements in the field of prognostic and/or predictive biomarkers for (Pediatric) IBD, at least at a research level. However, it will probably take at least the same amount of time, if not even longer, to implement a real translation to the clinical medicine on a large scale.

## Article Highlights

- Existing prognostic biomarkers in IBD are mainly based on disease behavior during the first months following diagnosis.
- To date no reliable endpoints have been validated as surrogates for the risk of disease complications.
- The development of disease prognostic biomarkers in IBD has been hampered by the lack of existing, quantifiable, validated outcome measures.
- Disease prognosis does not predict response to a specific therapy in IBD.
- Extensive phenotypic heterogeneity even within IBD sub-entities complicates the development of prognostic and predictive biomarkers.
- Systems biology provides an unbiased approach towards addressing variation in both omics and clinical datasets and has been successfully employed for the development of prognostic biomarkers in other diseases.
- The development of disease predictive biomarkers may provide a more realistic and equally beneficial alternative.
